# Effect of starvation on global gene expression and proteolysis in rainbow trout (*Oncorhynchus mykiss*)

**DOI:** 10.1186/1471-2164-8-328

**Published:** 2007-09-19

**Authors:** Mohamed Salem, Jeff Silverstein, Caird E Rexroad, Jianbo Yao

**Affiliations:** 1 Laboratory of Animal Biotechnology and Genomics, Division of Animal and Nutritional Sciences, West Virginia University, Morgantown, WV 26505, USA; 2 U.S. Department of Agriculture, Agricultural Research Service, National Center for Cool and Cold Water Aquaculture, Kearneysville, WV 25430, USA

## Abstract

**Background:**

Fast, efficiently growing animals have increased protein synthesis and/or reduced protein degradation relative to slow, inefficiently growing animals. Consequently, minimizing the energetic cost of protein turnover is a strategic goal for enhancing animal growth. Characterization of gene expression profiles associated with protein turnover would allow us to identify genes that could potentially be used as molecular biomarkers to select for germplasm with improved protein accretion.

**Results:**

We evaluated changes in hepatic global gene expression in response to 3-week starvation in rainbow trout (*Oncorhynchus mykiss*). Microarray analysis revealed a coordinated, down-regulated expression of protein biosynthesis genes in starved fish. In addition, the expression of genes involved in lipid metabolism/transport, aerobic respiration, blood functions and immune response were decreased in response to starvation. However, the microarray approach did not show a significant increase of gene expression in protein catabolic pathways. Further studies, using real-time PCR and enzyme activity assays, were performed to investigate the expression of genes involved in the major proteolytic pathways including calpains, the multi-catalytic proteasome and cathepsins. Starvation reduced mRNA expression of the calpain inhibitor, calpastatin long isoform (CAST-L), with a subsequent increase in the calpain catalytic activity. In addition, starvation caused a slight but significant increase in 20S proteasome activity without affecting mRNA levels of the proteasome genes. Neither the mRNA levels nor the activities of cathepsin D and L were affected by starvation.

**Conclusion:**

These results suggest a significant role of calpain and 20S proteasome pathways in protein mobilization as a source of energy during fasting and a potential association of the CAST-L gene with fish protein accretion.

## Background

Protein turnover is a major determinant in the conversion of feed into growth [1]. Young and others [2] reported that 15% to 25% of the energy consumed by growing animals is used for protein breakdown and re-synthesis. Consequently, minimizing the energetic cost of protein turnover is a strategic goal for enhancing animal growth and feed efficiencies.

Protein accretion is the net effect of protein synthesis and degradation. When protein synthesis rates are similar, factors affecting protein degradation are critical in explaining differences in growth efficiency of individuals [1,3]. Recently, we showed that fish muscle protein degradation depends on activities of proteolytic enzymes that are tightly controlled and regulated [4-7]. The major systems involved in fish muscle proteolysis are 1) membrane-bound lysosomal enzymes, 2) ubiquitin-proteasome pathway enzymes, and 3) calcium-dependent calpain proteinases.

Fish swiftly use proteins as oxidative substrates [8] and proteins have traditionally been considered to be the usual gluconeogenic precursors during starvation in fish [9]. Rates of protein synthesis also fall during starvation [10,11]. The primary objective of this study was to use microarray technology to identify genes/pathways involved in starvation-related protein turnover. These genes could potentially be used as molecular biomarkers to study protein turnover and select for germplasm with improved protein accretion in rainbow trout (*Oncorhynchus mykiss*).

Salmonids during their life cycle may face extended periods without food. Fish during starvation depends on body energy reserves. An effective way to identify the relationships between major metabolic pathways and body processes is to examine changes in metabolism during starvation. The secondary objective of this study was to use microarray to identify metabolic adaptations of liver tissue during starvation in rainbow trout.

Our microarray experiments showed a synchronized down-regulated expression of protein biosynthesis genes in starved fish but no significant changes of gene expression in the major protein catabolic pathways were observed. To further investigate the effect of starvation on protein degradation, we used real-time PCR and enzyme activity assays (more sensitive and accurate methods) to measure the expression of genes and enzyme activities in the major proteolytic systems. Our results suggest a significant role of calpain and 20S proteasome pathways in rainbow trout protein turnover under fasting condition.

## Results and discussion

**Figure 1:**
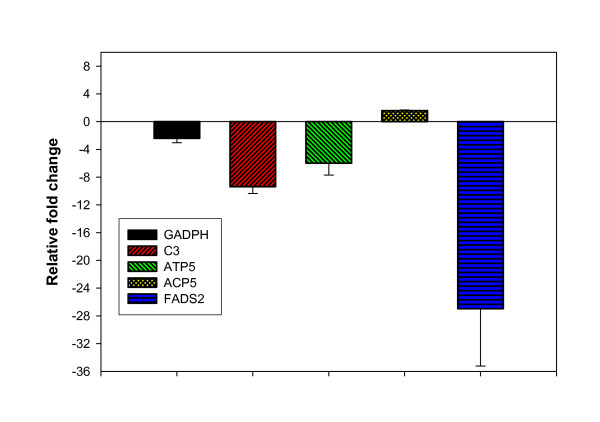
Quantitative real-time PCR confirmation of differential expression for 5 randomly selected genes identified by microarray as differentially expressed (Means ± S.E., n = 6, P < 0.05). The expression trends for all 5 genes were similar in both microarray and real-time PCR analyses. The average fold changes detected by microarray for GADPH, C3, ATP5, ACP5 and FADS2 were -2.9, -2.7, -5.0, 5.7, and -9.5, respectively.

**Figure 2:**
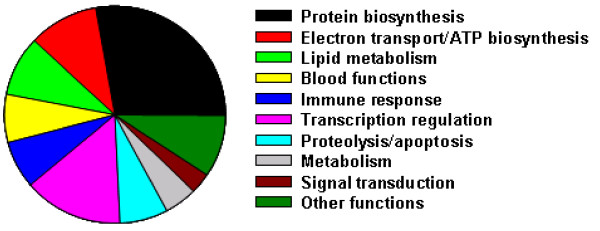
Summary of differentially expressed genes that have significant tBLASTX identity with genes assigned to GO biological function categories.

We performed microarray experiments to evaluate the changes in hepatic gene expression in response to starvation in rainbow trout. Microarray analysis defined 202 down-regulated and 27 up-regulated unique transcripts in starved fish (± 1.5-fold change, P < 0.05). Expression of 5 randomly selected genes, identified by microarray as differentially expressed, was confirmed by quantitative real time PCR analysis (Fig. 1, p < 0.05), indicating the reliability of the microarray data. The expression trends of all 5 genes were similar in both microarray and real-time PCR analyses. The differentially expressed genes were classified according to the GO biological functions (Fig. 2).

### Protein biosynthesis

Our microarray data showed that 28% of the differentially expressed genes belong to the protein biosynthesis machinery (Fig. 2). Almost all the protein biosynthesis genes were down-regulated (Table 1). The list includes 47 cytoplasmic ribosomal protein genes (30 for large subunit and 17 for small subunit) and 5 mitochondrial ribosomal protein genes (2 for large subunit and 3 for small subunit). Ribosomal proteins are essential for mRNA translation into protein [12], and they are integral components of the ribosome. Ribosomal proteins stabilize the rRNA structure and regulate translocation of mRNA and tRNA, which is necessary for optimal translation [13]. The down-regulated expression of the ribosomal proteins in starved fish suggests a well-regulated decrease in rate of protein synthesis at transcription. Control of gene expression at transcription would allow rapid suppression of protein biosynthesis when amino acids are deficient.

**Table 1:** Differentially expressed genes of the protein biosynthesis

**Symbol**	**Gene name**	**ACC.#**	**p value**	**GO Term**	**Fold change**
Mrpl36	Mitochondrial ribosomal protein L36	CB511228	7.8E-03	protein biosynthesis – GO:0006412	-4.1
Mrpl44	Mitochondrial ribosomal protein L44	CK991088	3.6E-04	protein biosynthesis – GO:0006412	-5.8
MRPS15	Mitochondrial ribosomal protein S15	CB494646	3.6E-03	protein biosynthesis – GO:0006412	-4.1
MRPS17	Mitochondrial ribosomal protein S17	CB504359	7.9E-03	protein biosynthesis – GO:0006412	-3.2
Mrps30	Mitochondrial ribosomal protein S30	CB492525	1.8E-04	protein biosynthesis – GO:0006412	-5.5
RPL10	Ribosomal protein L10	CA770456	4.7E-03	protein biosynthesis – GO:0006412	-3.3
RPL10A	Ribosomal protein L10a	CB491051	1.3E-03	protein biosynthesis – GO:0006412	-2.4
Rpl11	Ribosomal protein L11	CA052515	4.5E-03	protein biosynthesis – GO:0006412	-3.7
Rpl12	Ribosomal protein L12	CA044425	2.2E-03	protein biosynthesis – GO:0006412	-3.4
RPL13A	Ribosomal protein L13a	CA052724	1.1E-02	protein biosynthesis – GO:0006412	-2.8
RPL14	Ribosomal protein L14	CB493023	3.9E-03	protein biosynthesis – GO:0006412	-4.9
RPL17	Ribosomal protein L17	CA062149	5.7E-03	protein biosynthesis – GO:0006412	-2.9
RPL21	Ribosomal protein L21	CA044118	1.0E-02	protein biosynthesis – GO:0006412	-3.5
RPL23	Ribosomal protein L23	CA061476	4.9E-04	protein biosynthesis – GO:0006412	-4.5
RPL23A	Ribosomal protein L23a	CA052583	4.4E-03	protein biosynthesis – GO:0006412	-3.2
Rpl24	Ribosomal protein L24	CA051932	1.5E-02	protein biosynthesis – GO:0006412	-2.5
Rpl26	Ribosomal protein L26	CB497727	3.6E-02	protein biosynthesis – GO:0006412	-3.1
RPL27	Ribosomal protein L27	CA037570	1.0E-02	protein biosynthesis – GO:0006412	-5.4
Rpl28	Ribosomal protein L28	CB502045	4.1E-04	protein biosynthesis – GO:0006412	-5.1
RPL3	Ribosomal protein L3	CB487027	1.5E-02	protein biosynthesis – GO:0006412	-5.6
Rpl31	Ribosomal protein L31	CK990835	5.7E-05	protein biosynthesis – GO:0006412	-4.2
Rpl32	Ribosomal protein L32	CB494481	3.7E-03	protein biosynthesis – GO:0006412	-3.2
RPL36	Ribosomal protein L36	CA046196	3.3E-03	protein biosynthesis – GO:0006412	-5.7
RPL36A	Ribosomal protein L36a	CB499948	8.5E-04	protein biosynthesis – GO:0006412	-4.8
Rpl37	Ribosomal protein L37	CB500526	1.7E-02	protein biosynthesis – GO:0006412	-3.7
Rpl39	Ribosomal protein L39	CB508357	9.0E-04	protein biosynthesis – GO:0006412	-3.7
RPL5	Ribosomal protein L5	CK991320	1.8E-02	protein biosynthesis – GO:0006412	-2.0
RPL7	Ribosomal protein L7	CK991334	8.3E-04	protein biosynthesis – GO:0006412	-3.1
RPL9	Ribosomal protein L9	CB492853	1.2E-02	protein biosynthesis – GO:0006412	-2.8
Rps12	Ribosomal protein S12	CA769642	2.5E-03	protein biosynthesis – GO:0006412	-3.4
RPS16	Ribosomal protein S16	CB492970	5.8E-04	protein biosynthesis – GO:0006412	-5.5
RPS18	Ribosomal protein S18	CB498298	5.4E-03	protein biosynthesis – GO:0006412	-2.3
Rps21	Ribosomal protein S21	CA047151	6.5E-03	protein biosynthesis – GO:0006412	-3.5
Rps23	Ribosomal protein S23	CA042659	8.5E-04	protein biosynthesis – GO:0006412	-5.9
Rps24	Ribosomal protein S24	CA769405	2.3E-02	protein biosynthesis – GO:0006412	-2.1
RPS25	Ribosomal protein S25	CA050917	2.9E-03	protein biosynthesis – GO:0006412	-3.2
Rps26	Ribosomal protein S26	CA061718	2.6E-02	protein biosynthesis – GO:0006412	-3.2
Rps27	Ribosomal protein S27	CK990906	6.1E-04	protein biosynthesis – GO:0006412	-3.7
Rps3	Ribosomal protein S3	CA058850	5.8E-03	protein biosynthesis – GO:0006412	-2.3
Rps7	Ribosomal protein S7	CB504457	3.4E-03	protein biosynthesis – GO:0006412	-4.2
Rplp2	Ribosomal protein, large P2	CB506488	7.2E-05	translational elongation – GO:0006414	-4.5
RPLP0	Ribosomal protein, large, P0	CA045397	6.9E-03	protein biosynthesis – GO:0006412	-2.0
Rplp1	Ribosomal protein, large, P1	CB496672	6.0E-03	protein biosynthesis – GO:0006412	-3.7
LOC363753	Similar to 40S ribosomal protein S17	CK991333	3.0E-03	protein biosynthesis – GO:0006412	-4.0
LOC667731	Similar to 40S ribosomal protein S2	CB498057	4.1E-03	protein biosynthesis – GO:0006412	-2.0
LOC501619	Similar to 40S ribosomal protein S29	CK991092	3.9E-03	protein biosynthesis – GO:0006412	-6.0
LOC676999	Similar to 40S ribosomal protein S6	CB496987	1.8E-03	protein biosynthesis – GO:0006412	-2.7
LOC675647	Similar to 40S ribosomal protein S7 (S8)	CB492855	2.5E-02	protein biosynthesis – GO:0006412	-2.4
LOC666546	Similar to 60S acidic ribosomal protein P1	CB498269	2.1E-03	protein biosynthesis – GO:0006412	-3.8
LOC498135	Similar to 60S ribosomal protein L18	CA061879	3.7E-02	protein biosynthesis – GO:0006412	-2.7
MGC72957	Similar to 60S ribosomal protein L18a	CB496920	1.0E-02	protein biosynthesis – GO:0006412	-3.6
LOC436164	Similar to 60S ribosomal protein L7a	CK990919	1.0E-02	protein biosynthesis – GO:0006412	-2.6
RGD1309784	Similar to ribosomal protein L24-like	CB509952	2.0E-03	protein biosynthesis – GO:0006412	-2.8
EEF1A1	Eukaryotic translation elongation factor 1 alpha 1	CB491069	1.5E-03	translational elongation – GO:0006414	-5.4
EEF1B2	Eukaryotic translation elongation factor 1 beta 2	CB500560	1.8E-02	translational elongation – GO:0006414	-2.5
EIF2C1	Eukaryotic translation initiation factor 2C, 1	CA055479	1.2E-02	protein biosynthesis – GO:0006412	2.3
Eif2s2	Eukaryotic translation initiation factor 2, subunit 2 (beta)	CB499647	3.4E-04	protein biosynthesis – GO:0006412	3.5
BTD	Biotinidase	CB492660	2.7E-04	biotin metabolism – GO:0006768	-8.7
SEC61B	Sec61 beta subunit	CK991330	1.0E-02	protein targeting – GO:0006605	-2.7
PPIA	Peptidylprolyl isomerase A (cyclophilin A)	CK990970	3.6E-04	protein folding – GO:0006457	-4.8
Hsbp1	Heat shock factor binding protein 1	CB508758	1.5E-05	protein folding – GO:0006457	-4.7
LOC290549	Heat shock protein	CB494575	3.5E-02	protein folding – GO:0006457	-2.4
UBA52	Ubiquitin A-52 residue ribosomal protein fusion product 1	CB496916	2.7E-03	protein biosynthesis – GO:0006412	-3.7
Cog7	Component of oligomeric golgi complex 7	CA050633	2.2E-02	protein transport-GO:0015031	1.8

Starved fish exhibited up-regulated expression of eukaryotic translation initiation factor 2C, 1, and S2, Translation initiation factors are essential for protein synthesis [14]. A similar eukaryotic translation initiation factor (eIF2α) is a highly conserved adaptor to stress [15]. The upregulated expression of the eukaryotic translation initiation factors may be to keep the basal level of protein translation or as a consequence of starvation stress. On the other hand, the eukaryotic translation elongation factors 1 alpha, EEF1A1, and beta, EEF1B2, were down-regulated. Jurss and coworkers [16] reported that rainbow trout *in vitro *protein synthesis-stimulating activity is reduced by food deprivation and can be compensated by addition of elongation factors EF1 and/or EF2. Consequently, availability of eukaryotic translation factors, EF1 and EF2, may be a rate-determining point for rainbow trout protein synthesis. In addition, the biotinidase transcript, which catalyzes the release of biotin from biocytin, was also down regulated in starved fish. Theoretically, formation of translation initiation complexes may depend on biotin [17]. Expression of the peptidyl-prolyl, cis-trans, isomerase A (Cyclophilin A) declined in starved fish. This enzyme is ubiquitous and encodes essential step in protein folding and post-translational modification. In addition, the beta subunit of Sec61 protein, which is involved in the co-translational protein transport system [18], was down-regulated in starved fish suggesting impaired intracellular protein transport.

Tripathi and Verma [11] reported a reduction in an overall capacity for protein synthesis in starved catfish. Peragon and colleagues [9] found that, during starvation, total hepatic-protein and RNA contents decreased significantly, and the absolute protein-synthesis rate also fell. Pace and Manahan [19] reported that protein synthesis accounted for 16± 4% and 75± 11% of metabolism in unfed and fed sea urchin larvae, respectively. Protein synthesis accounts for a high percentage of the individual's metabolic costs. Consequently, regulation of protein synthesis is a promising means to limit energy expenditures under unfavorable feeding condition. These reports are consistent with our results showing highly regulated protein synthesis rates in fish liver in response to starvation. Less essential pathways such as protein synthesis are reduced to minimize ATP demands [20,21].

### Mitochondrial functions and glucose metabolism

Several genes belonging to complexes of the mitochondrial electron transport pathway were down-regulated. These genes include complex III (Ubiquinol-cytochrome c oxidoreductases), complex IV (Cytochrome c oxidase) and complex V ATP synthase (Table 2). In addition, succinyl-CoA synthetase alpha subunit of the citric acid cycle was down-regulated in starved fish. Furthermore, several members of the adenine nucleotide translocator that transports ATP out of the mitochondrion matrix in exchange for ADP produced in the cytosol were down-regulated. Similarly, a mitochondrial phosphate carrier, which returns phosphate generated by ATP breakdown to the mitochondrion, was down-regulated. An outer mitochondrial membrane translocase, TOMM7, which mediates the translocation of preproteins into or across the outer mitochondrial membrane, was down-regulated in starved fish. Conversely, TOMM40 was up-regulated. The vast majority of mitochondrial proteins are synthesized from nuclear DNA as precursor proteins on cytosolic polysomes, and these proteins must be imported into the mitochondria [22]. Our results point to impaired mitochondrial functions and an overall reduction in ATP production capacity as a result of starvation. Maintenance of high metabolic rates following the exhaustion of energy reserves during starvation would compromise animals' ability to survive [23].

**Table 2:** Differentially expressed genes of the mitochondrial functions and glucose metabolism

**Symbol**	**Gene name**	**ACC.#**	**p value**	**GO Term**	**Fold change**
UCRC	Ubiquinol-cytochrome c reductase complex	CA038364	2.0E-02	electron transport – GO:0006118	-4.4
CYBRD1	Cytochrome b reductase 1	CK990761	2.0E-02	electron transport – GO:0006118	-2.4
COX4I1	Cytochrome c oxidase subunit IV isoform 1	CK990997	5.2E-04	electron transport – GO:0006118	-3.3
COX6B1	Cytochrome c oxidase subunit Vib polypeptide 1 (ubiquitous)	CA047209	7.9E-03	electron transport – GO:0006118	-3.8
COX7A2	Cytochrome c oxidase subunit VIIa polypeptide 2 (liver)	CB511353	4.4E-02	electron transport – GO:0006118	-3.1
Cox6c	Cytochrome c oxidase, subunit VIc	CA044426	2.4E-03	electron transport – GO:0006118	-4.3
Cox17	Cytochrome c oxidase, subunit XVII assembly protein homolog	CB507314	3.4E-02	electron transport – GO:0006118	-2.2
	Similar to NP_008189.1 cytochrome c oxidase subunit II	CA768526	3.5E-03	electron transport – GO:0006118	-2.2
	Similar to NP_536845.1 cytochrome c oxidase subunit I	CB494005	2.0E-04	electron transport – GO:0006118	-3.1
	Similar to NP_006917.1 cytochrome c oxidase subunit I	CN442555	8.8E-03	electron transport – GO:0006118	-2.4
	Similar to NP_536846.1 cytochrome c oxidase subunit II	CN442553	4.3E-04	electron transport – GO:0006118	-6.0
ATP5G1	ATP synthase, H+ transporting, mitochondrial F0 complex, C1	CK991109	1.3E-02	ATP synthesis – GO:0015986	-2.5
ATP5J2	ATP synthase, H+ transporting, mitochondrial F0 complex, F2	CB493612	8.3E-03	ATP biosynthesis – GO:0006754	-5.0
Atp5l	ATP synthase, H+ transporting, mitochondrial F0 complex, g	CB497057	3.5E-03	ATP synthesis – GO:0015986	-3.3
ATP5B	ATP synthase, H+ transporting, mitochondrial F1 complex, beta	CK990869	1.7E-04	ATP synthesis – GO:0015986	-3.9
ATP6V1H	ATPase, H+ transporting, lysosomal 50/57kDa, V1 subunit H	CA053755	9.7E-03	ion transport – GO:0006811	-2.4
	Similar to mitochondrial ATP synthase epsilon chain	CB496505	8.2E-04	ATP biosynthesis – GO:0006754	-5.7
SUCLG1	Succinyl-CoA synthetase alpha subunit	CB498701	1.4E-02	succinate-CoA ligase (GDP-forming)-GO:0004776	4.2
Slc25a5	Mitochondrial carrier, adenine nucleotide translocator, member 5	CA058445	2.5E-02	transport – GO:0006810	-2.6
SLC25A6	Mitochondrial carrier; adenine nucleotide translocator, member 6	CK990577	2.2E-02	transport – GO:0006810	-2.4
SLC25A25	Mitochondrial carrier; phosphate carrier, member 25	CA042906	3.1E-03	transport – GO:0006810	-3.9
TOMM7	Translocase of outer mitochondrial membrane 7	CB497943	1.0E-03	protein transport – GO:0015031	-3.7
TOMM40	Translocase of outer mitochondrial membrane 40	CB498734	2.4E-04	lipid transporter activity – GO:0005319	5.4
GAPDH	Glyceraldehyde-3-phosphate dehydrogenase	CA050886	4.8E-03	GO:0003824 : catalytic activity	-2.9
RPIA	Ribose 5-phosphate isomerase A (ribose 5-phosphate epimerase)	CB503502	1.2E-02	pentose-phosphate shunt – GO:0009052	1.8
ST6GALNAC6	ST6 (alpha-N-acetyl-neuraminyl-2,3-beta-galactosyl-1,3)-N-acetylgalactosaminide alpha-2,6-sialyltransferase 6	CA050762	2.4E-02	carbohydrate metabolism – GO:0005975	-2.0
Fahd1	Fumarylacetoacetate hydrolase domain containing 1	CB492598	1.5E-02	metabolism – GO:0008152	-2.2
Tat	Tyrosine aminotransferase	CA056381	2.6E-02	amino acid and derivative metabolism – GO:0006519	-2.5
FUK	Fucokinase	CA040903	5.5E-04	fucokinase activity – GO:0050201	4.7
Ces2	Carboxylesterase 2	CA039230	4.3E-02	catabolism – GO:0009056	1.7
Ces6	Carboxylesterase 6	CB496876	2.8E-02	carboxylesterase activity – GO:0004091	1.7
SAT	Spermidine/spermine N1-acetyltransferase	CB488575	3.4E-03	diamine N-acetyltransferase activity – GO:0004145	3.8

On the other hand, energy production through anaerobic mechanisms seems to be less sensitive than aerobic (mitochondrial) mechanisms in responding to an inadequate supply of energetic compounds from food sources. Starvation caused a decreased expression of a single glycolytic pathway enzyme; GAPDH. Surprisingly, other members of the glycolytic pathway, including the highly regulated and rate-limiting enzymes, hexokinase, phosphofructokinase and pyruvate kinase were not affected (Table 2). Moreover, expression of ribose 5-phosphate isomerase A, a member of the pentose phosphate pathway that is responsible for 30% of the hepatic glucose oxidation [14], was up-regulated in starved fish. The lack of a coordinated, down-regulated expression of the glycolytic pathway enzymes upon starvation support the notion of altered regulation for carbohydrate metabolism in carnivorous fish such as rainbow trout as compared to mammals [23,24]. To explain the low dietary glucose utilization in rainbow trout, Wilson [24] hypothesized the existence of dysfunctional regulation of hepatic glycolysis and gluconeogenesis. No dietary requirement for carbohydrate has been demonstrated in fish. When carbohydrates are not provided in the diet, other nutrients such as protein and lipids are catabolized for energy [24]. The stabilized expression of most glycolytic pathway enzymes may be to preserve the enzymatic machinery of the gluconeogenesis. While the activities of most pathways are reduced during starvation, some pathways, such as gluconeogenesis, may remain unaltered or become enhanced [25,26] in vital tissues during the initial stages of fish starvation. Glucose is needed for the continued function of essential organs like brain. Another explanation for the maintenance of glycolytic enzymes' expression is short duration of feed deprivation (3 weeks) used in this experiment.

### The antioxidant systems

In starved fish, reduced expression of a number of transcripts related to maintenance of intracellular redox status was observed. These transcripts include 4 glutathione S-transferase (GST) transcripts and the glutathione peroxidase (GPX) gene (Table 3). These genes encode for antioxidants required to defend against reactive oxygen species (ROS) generated during the aerobic metabolic activities. GPX breaks down hydrogen peroxide [27] and GST conjugates reduced glutathione to xenobiotics or cellular components damaged by ROS [28]. Another down-regulated transcript is Hydroxyacid oxidase (Hao1) whose expression has been shown to be liver-specific and targeted to peroxisomes. Hao1 belongs to a family of enzymes that convert a broad range of α-hydroxy acids to α-keto acids and concomitantly reduce molecular oxygen to H_2_O_2 _[29]. Oxidative activities of mitochondria are a primary endogenous source of the reactive oxygen species (ROS). Consequently, we predict that, as the rate of ROS generation is decreased as a result of reduced aerobic metabolism in starved fish, the rate of the cellular antioxidants generation is decreased as well.

**Table 3:** Differentially expressed genes of the antioxidant system

**Symbol**	**Gene name**	**ACC.#**	**p value**	**GO Term**	**Fold change**
GSTO2	Glutathione S-transferase omega 2	CA064086	2.9E-03	metabolism – GO:0008152	-3.8
GSTP1	Glutathione S-transferase pi	CA050452	1.0E-03	glutathione transferase activity – GO:0004364	-7.1
GSTZ1	Glutathione transferase zeta 1 (maleylacetoacetate isomerase)	CB492382	6.9E-03	L-phenylalanine catabolism – GO:0006559	-4.5
MGST3	Microsomal glutathione S-transferase 3	CA061668	8.1E-03	lipid metabolism – GO:0006629	-5.3
GPX4	Glutathione peroxidase 4 (phospholipid hydroperoxidase)	CB510303	1.2E-02	phospholipid metabolism – GO:0006644	-3.1
HAO1	Hydroxyacid oxidase (glycolate oxidase) 1	CB502864	4.1E-02	fatty acid alpha-oxidation – GO:0001561	-2.4
TXN	Thioredoxin	CA057296	2.2E-05	electron transport – GO:0006118	-6.8
LOC389207	Similar to glutaredoxin cysteine-rich 1 protein	CB496770	7.3E-03	electron transport – GO:0006118	-3.0

Several members of the antioxidant systems that keep intracellular redox homeostasis including thioredoxin, glutaredoxin-like transcripts and disulfide reductases exhibited decreased expression in starved fish (Table 3). Jimenez and coworkers [30] reported that glucose deprivation reduced levels of thioredoxin-like protein. Conversely, its over-expression protects against glucose deprivation-induced cytotoxicity. Consequently, our results support the assertion that thioredoxin might be involved in cellular response to starvation stress.

### Lipid and prostaglandin metabolism

Fish use lipids as the major energy source in contrast to mammals that depend primarily on carbohydrates [31]. Microarray analysis revealed a decreased mRNA accumulation of the apolipoproteins including Apob, Apoa1, Apoc1, Apoc2, Apoe, and Apoh in starved fish (Table 4). Apolipoproteins are plasma lipoprotein complexes that are synthesized mainly in the liver, bind to lipids, and transport them to different tissues through circulation [32].

**Table 4:** Differentially expressed genes of the lipid metabolism

**Symbol**	**Gene name**	**ACC.#**	**p value**	**GO Term**	**Fold change**
APOB	Apolipoprotein B (including Ag(x) antigen)	CB511166	1.7E-02	lipid metabolism – GO:0006629	-4.1
Apoa1	Apolipoprotein A-I	CB510796	1.5E-03	lipid transport – GO:0006869	-3.0
Apoc1	Apolipoprotein C-I	CA037557	1.7E-03	transport – GO:0006810	-4.6
APOC2	Apolipoprotein C-II	CB496914	1.8E-02	lipid transport – GO:0006869	-2.4
Apoe	Apolipoprotein E	CB506103	2.3E-02	lipid transport – GO:0006869	-2.2
APOH	Apolipoprotein H (beta-2-glycoprotein I)	CB492833	1.3E-02	defense response – GO:0006952	-2.4
	Similar to NP_000473.1 apolipoprotein A-IV precursor	CB496971	1.7E-02	lipid transport – GO:0006869	-3.0
FABP1	Fatty acid binding protein 1, liver	CB509924	1.6E-03	fatty acid metabolism – GO:0006631	-5.0
Fabp3	Fatty acid binding protein 3, muscle and heart	CB507515	3.1E-02	phosphatidylcholine biosynthesis – GO:0006656	-2.1
RBP1	Retinol binding protein 1, cellular	CB492550	4.1E-04	vitamin A metabolism – GO:0006776	-4.5
RBP2	Retinol binding protein 2, cellular	CB496593	8.5E-04	vitamin A metabolism – GO:0006776	-4.5
FADS2	Fatty acid desaturase 2	CB494661	3.1E-04	fatty acid desaturation – GO:0006636	-9.5
Ptgds2	Prostaglandin D2 synthase 2	CA038730	1.1E-02	prostaglandin biosynthesis – GO:0001516	-2.7
ADFP	Adipose differentiation-related protein	CB514104	9.5E-03	lipid metabolism – GO:0006629	-3.5

Starved fish showed reduced mRNA accumulation of the fatty acid binding proteins, FABP1 and FABP3 and the retinol binding proteins, RBP1 and RBP2 (Table 4). FABPs and RBPs are collectively referred to as the intracellular lipid binding proteins. RBPs bind retinoids, which are essential for growth, vision, reproduction, hematopoiesis and immune function. FABPs play an important role in the intracellular uptake and transport of long-chain fatty acids through the aqueous cytoplasm to the site of their oxidation in the mitochondria or peroxisomes [33]. FABPs concentration increases with treatments that increase fatty acid metabolism, and it is positively correlated with the ability of tissues to metabolize fat [34]. Down-regulated expression of FABPs suggests reduced hepatic fatty acid metabolism that may contribute to the aforementioned overall decrease in mitochondrial ATP production in starved fish.

The microarray data did not reveal any elevated expression of the liver fatty acid oxidation mechanisms in starved fish suggesting that rainbow trout may mobilize fat from extra-hepatic resources to fuel metabolism during feed deprivation. Jeziersk and colleagues [35] reported that upon starvation, visceral lipid contributed the most to energy metabolism compared to muscle and liver fat depots. The absolute amount of fat derived from the liver was much smaller than that of muscle and viscera. Rasmussen and coworkers [36] reported that a 50% increase in feed lipid content enhanced fillet lipid levels by 20% and caused a 15–20% increase in the visceral fraction. Our previous results, using microarray and proximate analyses, did not reveal any significant modification of the lipolysis pathways in atrophying rainbow trout muscle [4,37]. Collectively, our results indicate that visceral fat is the first to be mobilized and perhaps the most important fat depot for energy in rainbow trout [35]. Liver and muscle lipids may be less mobile than visceral lipid. Detailed studies are needed to explore different mechanisms that regulate lipid mobilization from visceral, liver and muscle stores.

A significant decrease in mRNA accumulation of the fatty acid desaturase 2 gene (FADS2) was observed in starved fish (Fig. 1, Table 4). FADS2 is a terminal component of the liver lipogenic microsomal stearyl-CoA desaturase system that uses O_(2) _and electrons from reduced cytochrome b5 to catalyze the insertion of a double bond into a spectrum of fatty acyl-CoA substrates, including palmitoyl-CoA and stearoyl-CoA. The closely related desaturase, FADS1, is a key regulatory enzyme of unsaturated fatty acid biosynthesis [38]. Jezierska and coworkers [35] reported a decline in the hepatic percentage of the monoenoic fatty acid upon starvation of rainbow trout; whereas, the saturates remained relatively constant and polyunsaturates increased. Smith and colleagues [39] reported that monounsaturated fatty acids, 16:1n-7, 18:1n-9 and 18:1n-7, of the spiny lobster, *Jasus edwardsii *decreased with starvation. These results indicate diet-dependent adaptive shifts in fish relative fatty acid composition. Unsaturated fatty acids are needed for their unique physical properties in biological membranes [40]. Nevertheless, unsaturated fatty acids are synthesized at considerable energetic cost; approximately 10 ATP are used for each desaturation and 2-carbon elongation. Consequently, it may be advantageous to delay synthesizing these costly molecules until feeding is resumed.

Starved fish exhibited reduced mRNA accumulation of prostaglandin D2 synthase 2 (Ptgds2). Ptgds2 is the precursor of 15-deoxy-delta12-14-prostaglandin J2 (15d-PGJ2) that plays a critical role in fat cell differentiation, inducing the expression of adipocyte-specific genes and promoting the formation of mature, lipid-laden adipocytes [41]. In addition, adipose differentiation-related protein (ADFP) expression was reduced in starved fish. ADFP is associated with early stages of adipocyte differentiation and may play a critical role in regulating the formation, turnover and metabolic consequences of fat formation in mammalian extra adipose tissues [42]. Collectively, gene expression data, relative to lipid metabolism, suggest that the need to reduce metabolic energy costs has slowed down mechanisms of lipid and fatty acid synthesis, lipid binding and transportation and adipocyte differentiation. More detailed investigations on fish may add new insights into the molecular evolution of the mechanisms regulating lipogenesis and lipolysis processes and should become the objective of further studies.

### Blood function

Starvation reduced expression of many iron homeostasis and blood function-related genes including oxygen carrier hemoglobin (HB), alpha and beta; plasma iron transport protein, transferrin; and the iron storing protein, ferritin (Table 5). Heme binding protein 1 was up-regulated in starved fish. Transcripts of blood coagulation proteins, fibrinogen, plasminogen, thrombin receptor, and antithrombin (SERPINC1) were down-regulated in starved fish. Haptoglobin, which binds free HB leaking from red blood cells under pathological conditions to protect against its harmful oxidative effects [43], was also down regulated. In addition, the important cardiovascular and body fluid homeostasis gene, natriuretic peptide precursor A [44] was down-regulated in starved fish. Collectively, these data suggest that iron homeostasis functions are compromised in starved fish. Head kidney is the main hematopoietic tissue in fish [45], however, liver is a highly vascularized tissue. Consequently, the contradictory expression of the liver iron homeostasis transcripts may be due to the red blood cells entering the liver.

**Table 5:** Differentially expressed genes of the blood functions

**Symbol**	**Gene name**	**ACC.#**	**p value**	**GO Term**	**Fold change**
HBA1	Hemoglobin, alpha 1	CB500796	1.2E-04	oxygen transport – GO:0015671	-3.7
Hba-a1	Hemoglobin alpha, adult chain 1	CB497424	2.7E-04	oxygen transport – GO:0015671	-5.4
Hbb	Hemoglobin beta	CB498575	6.8E-04	oxygen transport – GO:0015671	-5.7
TF	Transferrin	CB496720	2.1E-02	iron ion transport – GO:0006826	-4.6
Hebp1	Heme binding protein 1	CA043780	8.9E-04	heme metabolism – GO:0042168	4.3
FTH1	Ferritin, heavy polypeptide 1	CB507396	1.3E-03	ferritin complex – GO:0008043	-4.9
F2R	Coagulation factor II (thrombin) receptor	CB493471	2.9E-02	blood coagulation – GO:0007596	-2.1
SERPINC1	Serpin peptidase inhibitor, clade C (antithrombin), member 1	CA038790	2.7E-03	blood coagulation – GO:0007596	-3.1
Fgg	Fibrinogen, gamma polypeptide	CA039531	5.1E-04	blood coagulation – GO:0007596	-3.1
Plg	Plasminogen	CA037954	7.4E-03	blood coagulation – GO:0007596	-3.9
HP	Haptoglobin	CB510638	7.1E-04	iron homeostasis – GO:0006879	-2.3
NPPA	Natriuretic peptide precursor A	BU965660	7.7E-03	blood pressure regulation – GO:0008217	-6.1
Hemt1	Hematopoietic cell transcript 1	CB512520	1.8E-03	protein folding – GO:0006457	-2.8
Narg1	NMDA receptor-regulated gene 1	CA063821	3.2E-03	angiogenesis – GO:0001525	2.3

### Immune response

Several immune-relevant genes were down-regulated in response to starvation (Table 6). The list includes 4 components of the complement system, C3, C5, CFB and CFP; 2 transcripts of C-type lectins, and the aforementioned transferrin, prostaglandin D2 synthase, glutathione peroxidases and hepatoglobin genes. These transcripts were identified by cDNA subtractive libraries as liver-made defense molecules and members of the fish innate immune system. These genes are inducible when fish are challenged with bacterial infection [46,47]. Furthermore, hepcidin, a potent antimicrobial peptide and important member of the fish innate immune system [48], was down-regulated, and transferrin, which is a positive acute phase protein in rainbow trout [49] was also down-regulated in starved fish. Transferrin, ferritin and hepcidin modulate iron homeostasis [50]; hence, they may control bacterial proliferation by limiting iron availability [51]. Down-regulation of the immune-relevant genes suggests mechanisms by which starved fish may demonstrate weakened pathogen resistance.

**Table 6:** Differentially expressed genes of the immune response

**Symbol**	**Gene name**	**ACC.#**	**p value**	**GO Term**	**Fold change**
C3	Complement component 3	CB514355	2.5E-03	inflammatory response – GO:0006954	-2.7
C5	Complement component 5	CB491279	2.8E-03	inflammatory response – GO:0006954	-4.0
CFB	Complement factor B	CB511435	1.9E-02	innate immune response – GO:0045087	-2.9
CFP	Complement factor properdin	CB498335	1.9E-03	complement activation – GO:0006957	-4.1
CLEC2B	C-type lectin domain family 2, member B	CB492852	8.4E-03	antimicrobial humoral response – GO:0019735	-8.5
	similar to C-type lectin-like receptor 2	CB496842	6.1E-04	antimicrobial humoral response – GO:0019735	-6.1
Hamp	Hepcidin antimicrobial peptide	CK991068	1.6E-03	iron homeostasis – GO:0006879	-3.4
Tcrb-V13	T-cell receptor beta, variable 13	CB491795	1.1E-04	T cell receptor signaling pathway – GO:0050852	3.9
TRA@	T cell receptor alpha locus	CB498619	2.2E-04	cellular defense response – GO:0006968	2.5
ACP5	Acid phosphatase 5, tartrate resistant	CB515428	2.8E-04	acid phosphatase activity – GO:0003993	5.7
VIPR1	Vasoactive intestinal peptide receptor 1	CB511922	4.4E-04	immune response – GO:0006955	-3.9
BANF1	Barrier to autointegration factor 1	CB505698	1.0E-03	response to virus – GO:0009615	-3.7
IGSF4C	Immunoglobulin superfamily, member 4C	CA040781	6.6E-05	immunoglobulin complex – GO:0019814	-3.3
LECT1	Leukocyte cell derived chemotaxin 1	CA037891	1.1E-03	proteoglycan metabolism – GO:0006029	-6.5
Edem1	ER degradation enhancer, mannosidase alpha-like 1	CA063288	3.4E-02	response to unfolded protein-GO:0006986	2.3
SERPING1	Serpin peptidase inhibitor, clade G (C1 inhibitor), member 1	CA037346	5.4E-03	immune response-GO:0006955/blood	1.5

On the other hand, few immune-relevant genes showed up-regulated expression in response to starvation (Table 6). Two transcripts belonging to the T cell receptor system that is involved in adaptive (lymphoid) immune responses [51] were up-regulated. In addition, starved fish exhibited increased expression of the tartrate-resistant acid phosphatase (ACP5) gene. Macrophages from mice over expressing ACP5 showed increased capacity for killing bacteria [52]. Lymphoid cells entering liver from circulation may be responsible for this change.

The immune response includes synthesis of potent antioxidants to protect cells against oxidative damage [53]. Starved fish exhibited reduced expression of several genes involved in managing oxidative stress, including glutathione *S*-transferases and glutathione peroxidases. These genes were consistently up-regulated in *Piscirickettsia salmonis*-infected macrophages of Atlantic salmon [51]. On the other hand, cytochrome P450 CYP1A1 and CYP3A43, which are components of the necessary detoxification pathway [54], were up-regulated in starved fish.

### Miscellaneous functions

Starved fish showed down-regulation of many transcripts classified into various functions including transcriptional housekeeping genes, transcription factors and regulators and genes belonging to several signal transduction pathways. A complete list of the differentially expressed genes is available at NCBI Gene Expression Omnibus (GEO) database with the accession number: GSE6944 [55].

### Proteolysis and amino acid metabolism

Fish swiftly use proteins as oxidative substrates [8]. Protein turnover is a crucial determinant in converting feed into growth [2]. During starvation, fractional protein-degradation rate increases significantly [9]. Protein degradation is a tightly controlled and regulated process that depends on at least three major proteolytic enzyme pathways [4,5]. Consequently, gene expression of these enzyme systems is expected to increase in starved fish. However, starved fish showed reduced expression of a number of transcripts related to amino acid and proteolysis functions (Table 7). Two proteasome and 3 ubiquitin transcripts were also down-regulated in starved fish.

**Table 7:** Differentially expressed genes of the proteolysis

**Symbol**	**Gene name**	**ACC.#**	**p value**	**GO Term**	**Fold change**
Psmc6	Proteasome (prosome, macropain) 26S subunit, ATPase, 6	CB518083	4.3E-02	protein catabolism – GO:0030163	-2.3
PSME2	Proteasome (prosome, macropain) activator subunit 2	CB491442	1.5E-02	protein catabolism – GO:0030163	-5.7
Ubc	Ubiquitin C	CA048031	9.6E-04	ubiquitin-dependent protein catabolism-GO:0006511	-2.8
UCHL5	Ubiquitin carboxyl-terminal hydrolase L5	CA053740	1.2E-02	ubiquitin-dependent protein catabolism-GO:0006511	-7.1
UBE1C	Ubiquitin-activating enzyme E1C (UBA3 homolog, yeast)	CA051243	4.6E-02	proteolysis – GO:0006508	-2.3
Ubb	Polyubiquitin	CB500037	6.5E-04	protein ubiquitination – GO:0016567 :	-4.4
FBXO38	F-box protein 38	CB509947	4.2E-04	ubiquitin cycle – GO:0006512	-7.0
RBX1	Ring-box 1	CA062272	8.6E-03	protein ubiquitination – GO:0016567	-3.8
TRIM39	Tripartite motif-containing 39	CB514566	2.6E-02	protein ubiquitination – GO:0016567	-2.0
PITRM1	Pitrilysin metallopeptidase 1	CA050901	7.5E-03	metalloendopeptidase activity-GO:0004222	-3.2
TIMP2	TIMP metallopeptidase inhibitor 2	CB492841	2.0E-02	enzyme inhibitor activity – GO:0004857	-4.0
ACY1	Aminoacylase 1	CA054045	2.1E-02	amino acid metabolism – GO:0006520	-2.1
ITIH5	Inter-alpha (globulin) inhibitor H5	CA039272	3.6E-02	hyaluronan metabolism – GO:0030212	-2.8
Itih3	Inter-alpha trypsin inhibitor, heavy chain 3	CA037372	1.8E-02	hyaluronan metabolism – GO:0030212	-2.8
NUPR1	P8 protein (candidate of metastasis 1)	CA043387	1.0E-02	induction of apoptosis – GO:0006917	-2.9
SQSTM1	Sequestosome 1	CB492474	1.8E-04	apoptosis – GO:0006915	-5.9
CASP10	Caspase 10, apoptosis-related cysteine peptidase	CB498395	7.4E-03	induction of apoptosis – GO:0006917	-3.0
PDCD6	Programmed cell death 6	CB514639	1.1E-03	apoptosis – GO:0006915	3.1
Rilp	Rab interacting lysosomal protein	CB517236	2.2E-02	transport – GO:0006810	-2.6
Edem1	ER degradation enhancer, mannosidase alpha-like 1	CA063288	3.4E-02	response to unfolded protein-GO:0006986	2.3

### Evaluation of protease gene expression using real-time PCR and enzyme activity assays

Since the microarray approach did not show significant changes of gene expression in protein catabolic pathways, we decided to use real-time PCR and the enzyme activity assays to measure the expression of genes and enzyme activities in the major proteolytic systems.

**Figure 3:**
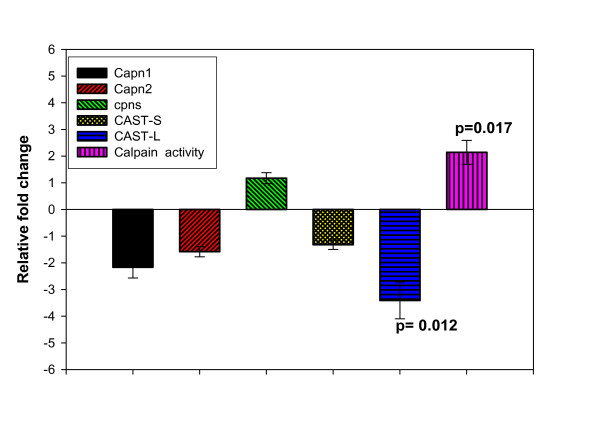
Effect of starvation on rainbow trout liver mRNA accumulation of the calpain catalytic subunits (Capn1 and Capn2), the regulatory subunit (cpns), the calpain inhibitors (CAST-L and CAST-S) and the corresponding calpain enzyme activity. Only significant p values are shown (Means ± S.E., n = 6).

As shown in Fig. 3, starvation did not affect the mRNA levels for Capn1, Capn2, cpns and CAST-S (p > 0.05). However, a significant decrease in CAST-L expression (p = 0.012), with a corresponding increase in the calpain catalytic activity, was observed in starved fish (Fig. 3, p = 0.017). Our results are consistent with a previous report showing that during starvation, activity of the calpain system in bovine skeletal muscle is controlled through decrease in expression of CAST [56].

CAST is a specific and the only known endogenous inhibitor of the calpain system. Mammalian experiments showed that β-adrenergic agonist administration decreases protein degradation through increased expression of the CAST gene [57]. CAST is associated with the muscle fractional degradation rate [58] and reduced muscle wastage in experimental animals [59]. Studies on the importance of CAST gene in fish are still limited. Previously, we reported that calpains play an important role in muscle proteolysis fueling metabolism of rainbow trout during starvation [6]. In addition, CAST-L and CAST-S mRNA were positively associated with muscle growth and firmness in rainbow trout [7]. The present results indicate that the calpain pathway may be involved in mobilizing hepatic proteins during starvation. The current study supports the importance of CAST gene in controlling fish protein turnover, and suggests that CAST-L may be a good candidate as a biomarker for fish protein accretion. Data collected recently in our lab indicated that the CAST-L and CAST-S genes are polymorphic in rainbow trout strains/crosses (unpublished data). More detailed studies to determine the physiological roles of CAST and the association of its polymorphisms with economically important traits in farmed fish are needed and are being currently conducted in our lab.

**Figure 4:**
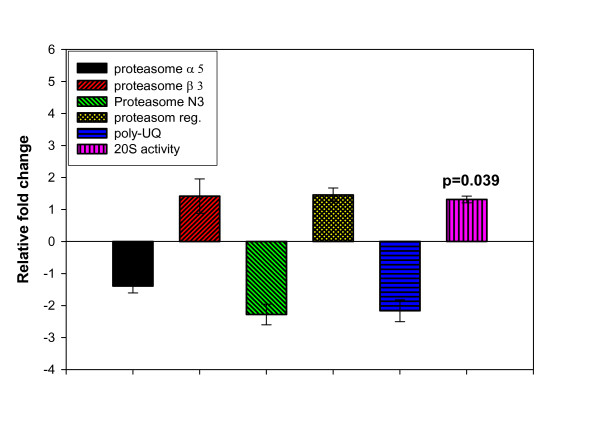
Effect of starvation on rainbow trout liver mRNA accumulation of the proteasome pathway genes (catalytic subunits alpha 5, beta 3 and N3, the regulatory subunit and polyubiquitin) and the corresponding enzyme activity of the 20S proteasome. Only significant p values are shown (Means ± S.E., n = 6).

Real time PCR analyses revealed no significant difference in expression of any of the studied proteasome genes including subunit alpha 5, subunit beta 3, the regulatory subunit 6, subunit N3 and poly-ubiquitin (Fig. 4C, p = 0.01). Unexpectedly, the corresponding 20S proteasome activity was slightly but significantly higher in starved fish (Fig. 4F, p = 0.039), suggesting a post-transcriptional regulatory effect of starvation on proteasome enzymatic activity. The ubiquitin-proteasome pathway is primarily responsible for proteolysis of normal mammalian muscle [60]. However, studies on fish indicate that the ubiquitin-proteasome proteolytic pathway is down-regulated in liver and muscle of starved rainbow trout without affecting mRNA of the proteasome N3 [61]. Our previous studies showed that the ubiquitin-proteasome system is not up-regulated during spawning-induced muscle proteolysis in rainbow trout [4,37]. Dobly and coworkers reported that proteasome activity in liver, but not in muscle, was negatively correlated with growth rates in rainbow trout [62]. These contradicting results suggest that our current observation of increased proteasome activity may represent a temporal change. Additional studies are needed to characterize the role of the proteasome system in fish protein accretion.

**Figure 5:**
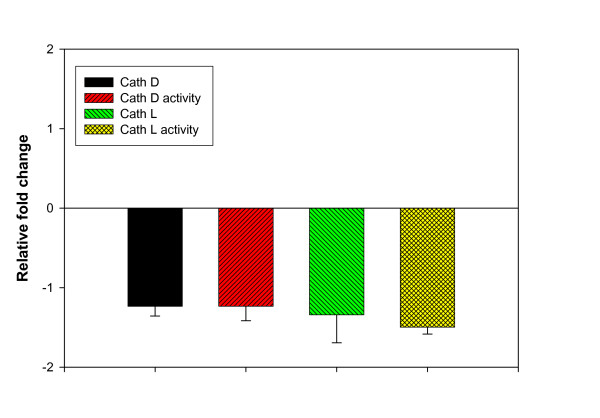
Effect of starvation on rainbow trout liver mRNA accumulation of cathepsins D and L and their corresponding enzyme activities. No significant difference was found (Means ± S.E., n = 6).

The mRNA abundance for cathepsins D and L and their corresponding enzyme activities were not affected by starvation as shown in Fig. 5 (p > 0.05). Our previous reports indicated that lysosomal cathepsins, particularly cathepsin-L, are the key proteases in spawning-induced proteolysis in rainbow trout [4,37]. In addition, we reported that β-adrenergic agonist administration reduced rainbow trout muscle cathepsin D activity [5]. The cathepsin pathway appears to play a major role in mobilizing muscle protein in salmonids, particularly when they cease feeding during their prolonged spawning migration [63]. Guderley found that, in Atlantic cod, hepatic contents of lysosomal proteases decreased with prolonged starvation, whereas in white muscle, starvation doubled specific activity of cathepsin D [27]. These results indicate that cathepsins may be less important in mobilizing hepatic proteins than in muscular proteins.

## Conclusion

The major responses of rainbow trout liver to starvation are: 1) a generalized decline in gene expression associated with a decrease in tissue metabolism, 2) an overall reduction in protein synthetic capacity, 3) impairment of mitochondrial (aerobic) ATP-biosynthetic functions while maintaining liver glycolytic/gluconeogenic competence, 4) down-regulated expression in mechanisms associated with hepatic lipid and fatty acid transport while maintaining fatty acid oxidation mechanisms, 5) significant increases in calpain and proteasome catabolic pathways, 6) fish may maintain tight-control on the mechanisms of protein metabolism more than lipid or carbohydrate under short term starvation condition and 7) extra-hepatic tissues, especially visceral fat, may play a major role in lipid metabolism upon starvation.

Maintenance of glycolytic expression may represent a short term effect (3 weeks) and suggest that normal turnover of energy reserves may be sufficient to maintain energy requirements during the initial stages of food deprivation. Nevertheless, the overall decrease of expression is indicative of a long-term metabolic response that aims to conserve energy reserves and enhance the ability to survive until feed is available. The use of microarray approach and enzyme activity measurements has allowed us to follow more closely the metabolic changes occurring during starvation. More detailed work is necessary to identify specific steps that control individual metabolic pathways and thereby determine energy use during starvation. The CAST-L gene is an appealing candidate as a potential biomarker for fish protein accretion. More detailed studies are needed to explore the physiological roles of the CAST-L gene in fish growth.

## Methods

### Fish and tissue sampling

The rainbow trout (*Oncorhynchus mykiss*) used in this study were from the National Center for Cool and Cold Water Aquaculture (NCCCWA) strain [64]. Prior to the study, the fish were reared under standard laboratory practices, and were fed to apparent satiation daily. A typical commercial trout feed was used (Zeigler Gold, Zeigler Bros. Inc, Garners, PA) with 42% crude protein and 16% crude fat. For both treatments six fish were each reared in a separate 10-liter tank for a total of 12 tanks. The fish were acclimated to the individual rearing units for 2 weeks prior to the study and all fish were feeding well at the initiation of the treatments. Water temperature was maintained at 14°C, and dissolved oxygen concentration was maintained close to saturation. Initial weight of the fish in the two treatments was not different (P > 0.37) at 193.0 ± 15.7 g. After 3 weeks the feed deprived fish weighed significantly less than the fed fish, 279.7 ± 19.8 g versus 172.0 ± 10.6 g. For the final weighing, fish were anesthetized one at a time with 0.1 mg/L MS-222. After weighing, a piece of liver was rapidly removed and placed in 1.5-ml test tubes, flash frozen in liquid nitrogen and then kept at -80°C until sample preparation. All animal handling and sampling procedures were reviewed and approved by the NCCCWA Institutional Animal Care and Use Committee.

### RNA preparation

Total RNA was isolated from each fish (6 fish/group) using Trizol reagent (Invitrogen, Carlsbad, CA) according to the manufacturer's instruction. Concentrations of isolated RNA were determined by measuring absorbance at 260 nm. The integrity of RNA was determined by agarose gel electrophoresis. Poly (A) mRNA was purified using Oligotex mRNA Mini Kit (Qiagen, Valencia, CA) according to the manufacturer's instruction.

### Microarrays, cDNA labeling and hybridization

A salmonid microarray containing cDNAs representing 16,006 genes selected from Atlantic salmon and rainbow trout expressed sequence tag databases [37,65] was used in the study. The microarray has been validated as a useful tool for rainbow trout studies [65]. A compete list of the genes on the array is available at the database of Consortium for Genomic Research on All Salmon Project [66]. Microarray slides were purchased from Dr. Ben Koop's laboratory at the University of Victoria. Arrays were performed on 4 independent fed and 4 independent fasted liver samples (4 biological replicates). Fluorophors (Cy3 and Cy5) were randomly assigned to RNA from each of the starved and fed fish to limit the dye effect. cDNA labeling and microarray hybridization procedures were essentially as we previously described [37,67]. Briefly, 0.4 μg of mRNA from each rainbow trout tissue was used as a template in reverse transcription reactions incorporating amino-allyl dUTP into the cDNA using oligo-d (T) primer and Superscript II reverse transcriptase (Invitrogen, Carlsbad, CA). The synthesized cDNAs from each starved and fed fish were differentially labeled using N-hydroxysuccinate-derived Cy3 or Cy5 dyes (GE Healthcare, Piscataway, NJ). Labeled cDNAs were purified using a PCR purification kit (Roche, Indianapolis, IN) to remove unincorporated dyes. The Cy3 and Cy5 labeled cDNAs were then combined and concentrated down to 20 μl using a Vacufuge vacuum concentrator (Eppendorf, Westbury, NY) followed by addition of 130 μl of Slidehyb 3 solution (Ambion, Inc. Austin, TX). Microarray hybridizations were performed on a Tecan HS400 automated microarray hybridization station (Tecan US, Durham, NC). The slides were placed on the machine at 60°C for 2 minutes followed by pre-hybridization at 55°C for 30 minutes with pre-hybridization solution (5 × SSC, 1% SDS, 1% BSA) under medium agitation. After a brief washing at 60°C for 1 minute, differentially labeled cDNAs in hybridization buffer (~145 μl) were injected into the hybridization chamber. The hybridizations were carried out for 3 hours at 60°C followed by another 13 hours at 55°C. Arrays were washed twice in 2 × SSC, 01% SDS, followed by twice in 0.1 × SSC, 0.1% SDS at room temperature. Following 2 more washes in 0.1 × SSC, the slides were rinsed in water and dried by centrifugation.

### Microarray scanning and data analysis

ScanArray Lite^® ^microarray scanner was used to scan arrays and ScanArray Express^® ^software (PerkinElmer, Wellesley, MA) was used to process array images, align spots, integrate robot-spotting files with the microarray images and quantify spots as we previously described [37]. Pre-processing and normalization of data were accomplished using the R-project statistical environment [68] and Bioconductor [69] through the GenePix AutoProcessor (GPAP) [70]. Data were pre-processed by: 1) Removing data points where signal intensities in both channels were less than a baseline threshold value of 200, 2) Calculating and subtracting local background fluorescence values from all feature intensities, 3) Log2-transforming the background subtracted Cy3/Cy5 ratios, 4) Calculating means of intensities within and across biological replicates, and 5) Defining spots that are larger or smaller than 2 standard deviations from the mean as outliers and eliminating them from calculation of the final means of Log2 ratios within and across the biological replicate arrays. All hybridizations were also subjected to manual review to ensure flagging and exclusion of all unacceptable spots. Following pre-processing, the expression results were normalized using global LOWESS normalization to adjust and balance individual signal intensities to reduce any systematic or technical variations. Diagnostic box plots of LOWESS normalized Log2 ratios were used to ensure that biological replicate arrays were similar in range. For each spot, t-statistic, P-value (probability), and fold change were calculated. Spots with one and half-fold change or more were considered differentially expressed using p value < 0.05. Four experiments were conducted. Microarray data were deposited (according to Microarray Gene Expression Data Society Standards) in the NCBI Gene Expression Omnibus with the series accession number: GSE6944 [55].

### Quantitative real-time PCR analysis

Quantitative real time PCR was used to confirm the expression of 5 differentially expressed genes identified by microarray experiments. In addition, real time PCR was also used to measure the mRNA levels of genes pertinent to 1) the calpain/calpastatin proteinase pathway [6,7] including the catalytic subunits of μ-calpain (Capn1) and m-calpain (Capn2), the calpain regulatory subunit (cpns), the calpastatin long isoform (CAST-L) and the calpastatin short isoform (CAST-S), 2) the proteasome multicatalytic pathway including proteasome subunits alpha 5, beta 3, N3, regulatory subunit 6, and the poly ubiquitin gene, 3) the cathepsin proteolytic pathway including cathepsins D and L [4,5]. Total RNA, isolated from liver samples (n = 6/group) using Trizol reagent, was further purified using an RNA clean-up kit according to the manufacturer's protocol (Zymo Research Corporation, Orange, CA). Two μg of each RNA sample were converted to cDNA using Superscript II reverse transcriptase (Invitrogen, Carlsbad, CA). To ensure RNAs were free of genomic DNA, negative control cDNAs were prepared by reverse transcription reactions without adding the reverse transcriptase. Real time PCR primers were designed based on each gene sequence (Table 8) using Primer3 software [71]. Quantitative PCR was performed in duplicate for each cDNA sample on a Bio-Rad iCycler iQ Real-Time PCR Detection System using iQ™ SYBR^® ^Green Supermix (Bio-Rad, Hercules, CA) in 25-μl reaction volumes containing 300 nM of each primer and cDNA derived from 0.2 μg of total RNA. The rainbow trout β-actin gene (its expression was not affected by starvation as shown by microarray analysis) was chosen as an endogenous control for normalization of the real time PCR analysis. Standard curves for each gene and the endogenous control were constructed using 10-fold serial dilutions of the corresponding plasmid. Standard curves were run on the same plate with the samples. Threshold lines were adjusted to intersect amplification lines in the linear portion of the amplification curve and cycles to threshold (Ct) were recorded. For each sample, the amount of target gene and endogenous reference was determined from the appropriate standard curve. The amount of the target gene was divided by the amount of reference gene to obtain a normalized target value. Mean differences in gene expression levels were determined by t-test and reported as relative fold changes.

**Table 8:** Primers used for real time RT-PCR analysis

**Gene name**	**Forward primer**	**Reverse primer**	**GenBank Acc. No./TIGR TC**
Capn1	5-GCCAAAACATTGCCTGTTATCTTAG-3	5-ATAGGAGGCCGTATCAAAATTCC-3	AY573919
Capn2	5-GATTCATCCAGAACGTGTAGG-3	5-GGTTAAACACTGGAGCGTGTC-3	AY573920
Cpns	5-GCTGCCTTCAAATCTGCATGT-3	5-TGTACCTGCGAGCGATCAACT-3	AF482696
CAST-S	5-ATGACAGAGCAGCTGTCCAATC-3	5-TGTTGAAGCAACATCACTGCAA-3	AY937408
CAST-L	5-ACGGCACCTTTCCTTTCCATTACCA-3	5-CGGGGGGAGCAGGAGACTTGGT-3	AY937407
Proteasome α5	5-GGTGTAGCGCTTCTCTTTGG-3	5-ACTGGACAAAGGTGCCTGAT-3	TIGR database TC78609
Proteasome β3	5-CCCATGGTGACAGAGGACTT-3	5-TGTCTGGCTCCCAGAGAGAT-3	TIGR database TC87448
Proteasome N3	5-AAGTGAACGACAGCACCATTC-3	5-CCTCATCGATCACCATCTGTT-3	CA386652
Proteasome Regulatory 6 5-CCGACCTCAGAGAAAAGGTG-3		5-AGAAGAGGTACTGGCGGACA-3	TIGR database TC87921
Plyubiqitin	5-CTGGAAGATGGTCGCACTCT-3	5-GATCTGCATACCTCCCCTCA-3	AF361365
Cathepsin-D	5-GCCTGTCATCACATTCAACCT-3	5-CCACTCAGGCAGATGGTCTTA-3	U90321
Cathepsin L	5-TGAAGGAGAAGATGTGGATGG-3	5-TTCCTGTCTTTGGCCATGTAG-3	AF358668
GADPH	5-CTGAACGACCACTTCGTCAA-3	5-TTACTCCTTGGTGGCCATGT-3	CB491826
C3	5-CTAACGAGGGCAAGCTCAAC-3	5-GCCTCCAGAGTGAGAAGGTG-3	CB514355
ATP5J2	5-GGGCGGATAAAAAGGCTAAT-3	5-GCCATTTATTGCCTGAAGGA-3	CB493612
ACP5	5-CAAGCAGTTCGACTGGATCA-3	5-ACCAATGGACCACACAGGAT-3	CB515428
FADS2	5-GTCCGTGCTTTGTGTGAGAA-3	5-TCAGAGACCCGACAACATCA-3	CB494661
β-Actin	5-GCCGGCCGCGACCTCACAGACTAC-3	5-CGGCCGTGGTGGTGAAGCTGTAAC-3	AJ438158

### Calpain activity assay

Calpain activity was measured using the calpain activity assay kit (Calbiochem, San Diego, CA) as described [4,5,7]. Briefly, liver tissues were homogenized in sample buffer. Cell lysates were incubated with the fluorogenic substrate Suc-LLVY AMC together with activation buffer. The release of the free AMC was measured at excitation and emission wavelengths of 370-nm and 450-nm, respectively. The change in proteasome activity was normalized to sample protein concentration and expressed as relative fluorescence fold change.

### Proteasome activity assay

Rainbow trout liver tissues were homogenized in lysis buffer (50 mM Tris pH 8.0, 0.1 mM EDTA, 1.0 mM 2β-mercaptoethanol) at 4°C, followed by centrifugation at 20,000 g for 10 min, and the supernatant was retained. The concentration of proteins was determined using BSA as standard. Proteasome peptidase activity was measured using the 20S proteasome assay kit as previously described [4,5] and according to the manufacturer's protocol (Alexis Biochemicals, San Diego, CA). The activity was measured using Suc-Leu-Leu-Val-Try-AMC as a substrate in a reaction mixture of 939 μl of 1 × reaction buffer, 10 μl of 3% SDS, 1 μl of 1000 × substrate solution and 50 μl of sample. Release of the fluorogenic reagent AMC was determined at excitation and emission wavelengths of 380-nm and 460-nm, respectively. The change in proteasome activity was normalized to sample protein concentration and expressed as relative fluorescence fold change.

### Cathepsins activity assay

Cathepsin-L activity was measured using the synthetic substrate Z-Phe-Arg-AMC essentially according to [4,5] as described in the manufacturer's protocol (InnoZyme™ Cathepsin-L Fluorogenic Activity Kit, Calbiochem, San Diego, CA). The release of the fluorogenic reagent AMC (7-amido-4-methylcoumarin) was determined by measuring fluorescence at excitation and emission wavelengths of 380-nm and 460-nm, respectively, in a Cary Eclipse fluorometer (Varian, Inc., Palo Alto, CA). Purified cathepsin-L and cathepsin-L inhibitor (kit supplied) were used for positive and negative controls, respectively. Activity of cathepsin-D was measured as described previously [4,5] using Bz-Arg-Gly-Phe-Phe-Pro-4MeOβNA, HCl (Calbiochem, San Diego, CA) as substrate. The reaction mixture contained 400 μl of 50 mM sodium acetate buffer, pH 4.0, 100 μl of 200 μM substrate solution and 40 μl of sample. The release of the fluorogenic reagent 4-MeOβNA was measured at excitation and emission wavelengths of 380-nm and 425-nm, respectively. The change in activity was normalized to the sample protein concentrations and expressed as relative fluorescence fold change.

### Statistical analyses

One-way analysis of variance (ANOVA) was performed on mean gene expression levels or enzyme activities using SigmaStat (version 3.11) software (Aspire Software International, Leesburg, VA). When one-way ANOVA showed significant effects, multiple mean comparisons were made using the Holm-Sidak method.

## Abbreviations

NCCCWA, National Center for Cool and Cold Water Aquaculture; GEO, Gene Expression Omnibus; Capn1, Catalytic subunits of μ-calpain; Capn2, Catalytic subunits of m-calpain; cpns, Calpain regulatory subunit; CAST-L; Calpastatin long isoform; CAST-S, Calpastatin short isoform; GADPH, Glyceraldehyde-3-phosphate dehydrogenase; C3, Complement component 3; ATP5J2, ATP synthase; ACP5, Acid phosphatase 5, tartrate resistant; FADS2, Fatty acid desaturase 2.

## Competing interests

The author(s) declares that there are no competing interests.

## Authors' contributions

MS was responsible for generating the gene expression and enzyme activity data, statistical analysis, and drafted the manuscript. JS co-authored the manuscript and was responsible for design of the starvation experiment and tissue collection. CR participated in coordination and provided some clones used in real time PCR analysis. JY was responsible for project development and is the corresponding author. All contributing authors reviewed and approved the final copy of this manuscript.
